# Effectiveness of high-flow nasal cannula in the management of acute hypercapnic respiratory failure: A meta-analysis of randomized controlled trials

**DOI:** 10.1097/MD.0000000000047099

**Published:** 2026-01-30

**Authors:** Cong Chen, Yanfang Peng, Jiahui Li, Shibin Peng

**Affiliations:** a Department of Intensive Care, The Second People’s Hospital, Chengdu, Sichuan Province, China.

**Keywords:** acute hypercapnic respiratory failure, high-flow nasal cannula, intubation, meta-analysis, mortality, non-invasive ventilation, PaCO_2_

## Abstract

**Background:**

Acute hypercapnic respiratory failure (AHRF) commonly complicates chronic respiratory diseases and is traditionally managed with noninvasive ventilation (NIV), although tolerance and interface-related limitations may reduce adherence. High-flow nasal cannula (HFNC) has been proposed as an alternative, but comparative efficacy in AHRF remains uncertain.

**Methods:**

We conducted a systematic review and meta-analysis in accordance with the Preferred Reporting Items for Systematic Reviews and Meta-Analyses. PubMed, Cochrane Library, Web of Science, and Embase were searched from inception to September 16, 2025, for randomized controlled trials comparing HFNC versus NIV in adults with AHRF. Risk of bias was assessed using Cochrane RoB 2.0. Pooled effects were summarized as risk ratios (RRs) for dichotomous outcomes and standardized mean differences (SMD) for continuous outcomes using fixed- or random-effects models according to heterogeneity.

**Results:**

Six randomized controlled trials (sample sizes 30–168) were included. HFNC and NIV showed no significant differences in partial pressure of carbon dioxide (SMD = −0.22, 95% confidence interval [CI]: −0.45 to 0.01; *I*^2^ = 20%), arterial pH (SMD = −0.03, 95% CI: −0.28 to 0.22; *I*^2^ = 45.2%), intubation (RR = 0.87, 95% CI: 0.41–1.82; *I*^2^ = 0%), or mortality (RR = 0.85, 95% CI: 0.47–1.56; *I*^2^ = 0%). Egger’s test suggested no significant publication bias.

**Conclusion:**

In adult AHRF, HFNC demonstrated efficacy comparable to NIV for gas-exchange and short-term clinical outcomes. HFNC may be considered an alternative when NIV is not tolerated, with explicit monitoring and timely escalation protocols.

## 1. Introduction

Acute hypercapnic respiratory failure (AHRF) is a critical condition characterized by elevated partial pressure of carbon dioxide (PaCO_2_) tension and respiratory acidosis resulting from inadequate alveolar ventilation. It commonly occurs in patients with underlying chronic respiratory diseases such as chronic obstructive pulmonary disease (COPD), obesity hypoventilation syndrome, and neuromuscular disorders.^[1]^ The management of AHRF requires prompt interventions to improve gas exchange, alleviate the work of breathing, and prevent endotracheal intubation and invasive mechanical ventilation, which are associated with high morbidity and mortality. Noninvasive respiratory support has therefore become a cornerstone of treatment, with 2 primary modalities currently in clinical use: noninvasive ventilation (NIV) and high-flow nasal cannula (HFNC). NIV, particularly bilevel positive airway pressure, has long been considered the standard of care for AHRF.^[2,3]^ randomized controlled trials (RCTs) and clinical guidelines have consistently demonstrated its ability to reduce the need for intubation, improve arterial blood gases, and decrease in-hospital mortality. Despite these benefits, NIV is not without limitations. Patient intolerance due to mask discomfort, air leaks, claustrophobia, and impaired communication can significantly reduce adherence. Moreover, inappropriate application or delayed recognition of NIV failure may lead to worsened outcomes, including increased mortality risk.^[4,5]^

In recent years, HFNC has emerged as a promising alternative to conventional NIV. By delivering heated, humidified oxygen at high flow rates through a nasal interface, HFNC provides several physiological advantages. These include the generation of a low level of positive end-expiratory pressure, reduction of anatomical dead space through carbon dioxide washout, improved mucociliary clearance, and better patient comfort compared with facemask interfaces. HFNC has demonstrated clinical efficacy in hypoxemic respiratory failure, with evidence supporting its role in reducing intubation rates and improving patient tolerance. However, its role in the management of hypercapnic respiratory failure remains less well defined and is an area of ongoing debate.^[6,7]^ Comparative studies examining HFNC and NIV in AHRF have yielded conflicting results. Some investigations suggest that HFNC may provide comparable efficacy to NIV in improving gas exchange, with superior patient comfort and fewer complications. Other studies, however, indicate that NIV remains superior for reducing PaCO_2_ and avoiding treatment failure, particularly in patients with moderate-to-severe acidosis. Given these inconsistencies, clinical decision-making remains challenging, and practice varies widely across institutions.^[8-10]^

The present meta-analysis aims to systematically review and quantitatively assess existing literature comparing HFNC with NIV in patients presenting with AHRF. By pooling data from RCTs, this analysis seeks to provide a more precise estimate of their relative effects on key clinical outcomes. The findings are expected to inform clinical practice and contribute to guideline development for the optimal noninvasive management of AHRF.

## 2. Materials and methods

### 2.1. Search strategy

This study was approved by the Ethics Committee of The Second People’s Hospital, Chengdu East New Area. This systematic review and meta-analysis was conducted in accordance with the Preferred Reporting Items for Systematic Reviews and Meta-Analyses (PRISMA) guidelines.^[11]^ A comprehensive literature search was performed in PubMed, The Cochrane Library, Web of Science, and Embase from their inception to September 16, 2025 to identify RCTs comparing HFNC with NIV in the treatment of AHRF. No language restrictions were applied, and studies published in non-English languages with available English abstracts were considered eligible. To ensure completeness, the reference lists of all retrieved articles, relevant reviews, and other sources were also manually screened. The search strategy combined subject headings (e.g., MeSH and Emtree terms) with free-text keywords, including but not limited to “high flow nasal cannula,” “HFNC,” “high flow oxygen therapy,” “heated humidified high flow nasal cannula,” “noninvasive ventilation,” “NIV,” “noninvasive positive pressure ventilation,” “NIPPV,” “BiPAP,” “CPAP,” “acute hypercapnic respiratory failure,” “AHRF,” “hypercapnia,” and “acute respiratory failure.”

### 2.2. Inclusion criteria and exclusion criteria

Studies were considered eligible if they met the following inclusion criteria: RCTs that compared HFNC with NIV in the treatment of AHRF; adult patients (≥18 years) diagnosed with AHRF, regardless of underlying etiology; studies that reported at least 1 relevant clinical outcome, such as treatment success or failure, intubation rate, mortality, arterial blood gas parameters, or patient tolerance; and full-text articles available in peer-reviewed journals. In this meta-analysis, treatment success was defined as clinical improvement without the need for escalation to invasive mechanical ventilation or crossover to an alternative noninvasive modality, while treatment failure was defined as persistent or worsening respiratory acidosis, deterioration in gas exchange, or the need for intubation or rescue therapy as determined by the study protocol.

Exclusion criteria were as follows: studies that were not RCTs, including observational studies, case series, case reports, reviews, and conference abstracts; studies involving pediatric patients or patients with chronic stable hypercapnia without acute decompensation; trials in which HFNC or NIV was used solely in postoperative, perioperative, or prophylactic settings rather than for AHRF; duplicate publications or secondary analyses of previously published trials with overlapping populations; and studies lacking sufficient data for extraction or statistical analysis.

### 2.3. Literature screening and data extraction

Two reviewers independently screened all retrieved studies in accordance with the predefined inclusion and exclusion criteria. Titles and abstracts were first assessed to exclude clearly irrelevant records, and the full texts of potentially eligible studies were subsequently reviewed to determine final eligibility. Data extraction was then performed independently by the same 2 reviewers, with results cross-checked for consistency. Any discrepancies were resolved through discussion, and when necessary, a third reviewer was consulted to achieve consensus. The following information was extracted from each included study: first author, year of publication, country, study setting, sample size, underlying diagnoses of enrolled patients, data collection methods, and follow-up duration. In addition, patient outcome measures were extracted, including arterial pH, PaCO_2_, intubation rate, and mortality.

### 2.4. Quality assessment

The risk of bias of the included studies was independently assessed by 2 reviewers using the Cochrane Risk of Bias 2.0 (RoB 2.0) tool for RCTs.^[12]^ The assessment covered the following domains: randomization process, deviations from intended interventions, missing outcome data, measurement of outcomes, and selection of reported results. The reviewers cross-checked their evaluations, and any discrepancies were resolved through discussion. When consensus could not be reached, a third reviewer was consulted to make the final judgment.

### 2.5. Statistical analyses

All statistical analyses were conducted using Review Manager (RevMan) version 5.4 and Stata version 18.0. Pooled estimates were calculated using either a fixed-effect model or a random-effects model, depending on the degree of heterogeneity among studies. Statistical heterogeneity was assessed with Cochran’s *Q* test and quantified using the *I*^2^ statistic, with values <25% indicating low heterogeneity, 25% to 50% indicating moderate heterogeneity, and >50% indicating substantial heterogeneity. When substantial heterogeneity was observed, a random-effects model was applied; otherwise, a fixed-effect model was used. For dichotomous variables, pooled results were expressed as risk ratios (RRs) with 95% confidence intervals (CIs). For continuous variables, pooled results were reported as mean differences with 95% CIs; if different measurement scales were used across studies, standardized mean differences were calculated. Publication bias was evaluated using Egger’s linear regression test, with a *P*-value < .05 considered indicative of significant bias. Sensitivity analyses were performed to assess the robustness of the pooled estimates by excluding studies judged to have a high risk of bias and comparing results derived from fixed-effect and random-effects models. The overall findings remained consistent, confirming the stability of the conclusions. All statistical tests were 2-sided, and a *P*-value < .05 was regarded as statistically significant.

## 3. Results

### 3.1. Search results and study selection

**Figure 1.**
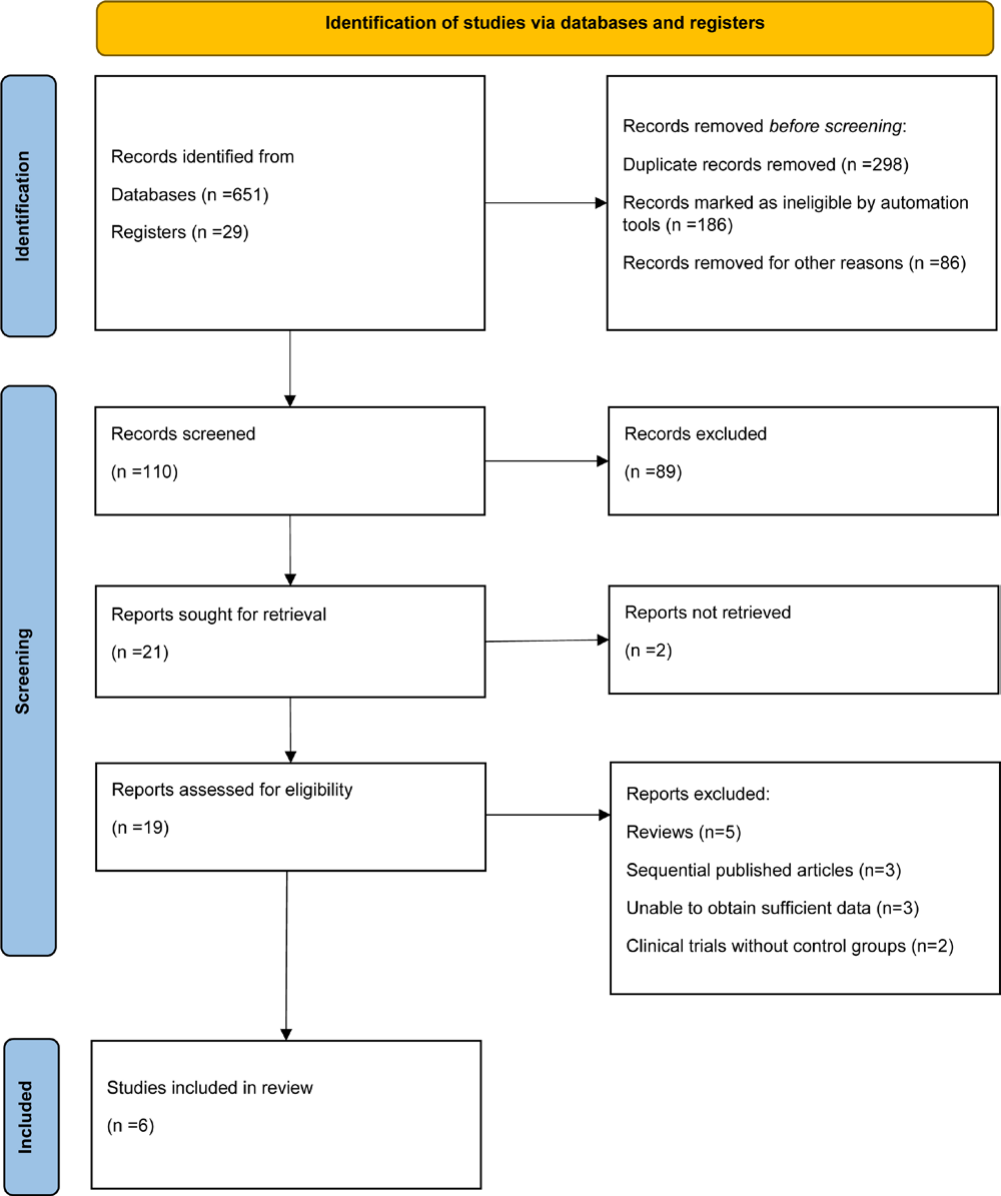
PRISMA flow diagram of study selection showing the process of identification, screening, eligibility assessment, and inclusion of randomized controlled trials. PRISMA = Preferred Reporting Items for Systematic Reviews and Meta-Analyses.

A total of 680 records were identified through database searching (n = 651) and trial registers (n = 29). After removing 298 duplicates, 186 records were excluded by automation tools, and 86 were removed for other reasons, leaving 110 records for screening. Following title and abstract screening, 89 records were excluded, and 21 reports were sought for full-text retrieval. Of these, 2 reports could not be retrieved. Nineteen reports were assessed for eligibility, among which 5 were excluded as reviews, 3 as sequentially published articles, 3 due to insufficient data, and 2 clinical trials without control groups. Ultimately, 6 RCTs met the inclusion criteria and were included in the final meta-analysis (Fig. 1).^[13-18]^

### 3.2. Study characteristics

The included RCTs were conducted across diverse clinical settings, including emergency departments, intensive care units, and specialized respiratory wards or centers. Sample sizes ranged from 30 to 168 participants. The majority of enrolled patients were diagnosed with COPD, with several studies focusing on patients presenting with concurrent acidosis. Other reported baseline conditions included cardiogenic pulmonary edema or congestive heart failure, as well as patients in the post-extubation phase. Data collection primarily involved physiological parameters and arterial blood gas measurements, with some studies additionally assessing work of breathing scores, patient comfort, and satisfaction levels. The duration of follow-up varied considerably across studies, ranging from 150 minutes to 5 days.

### 3.3. Results of quality assessment

**Figure 2.**
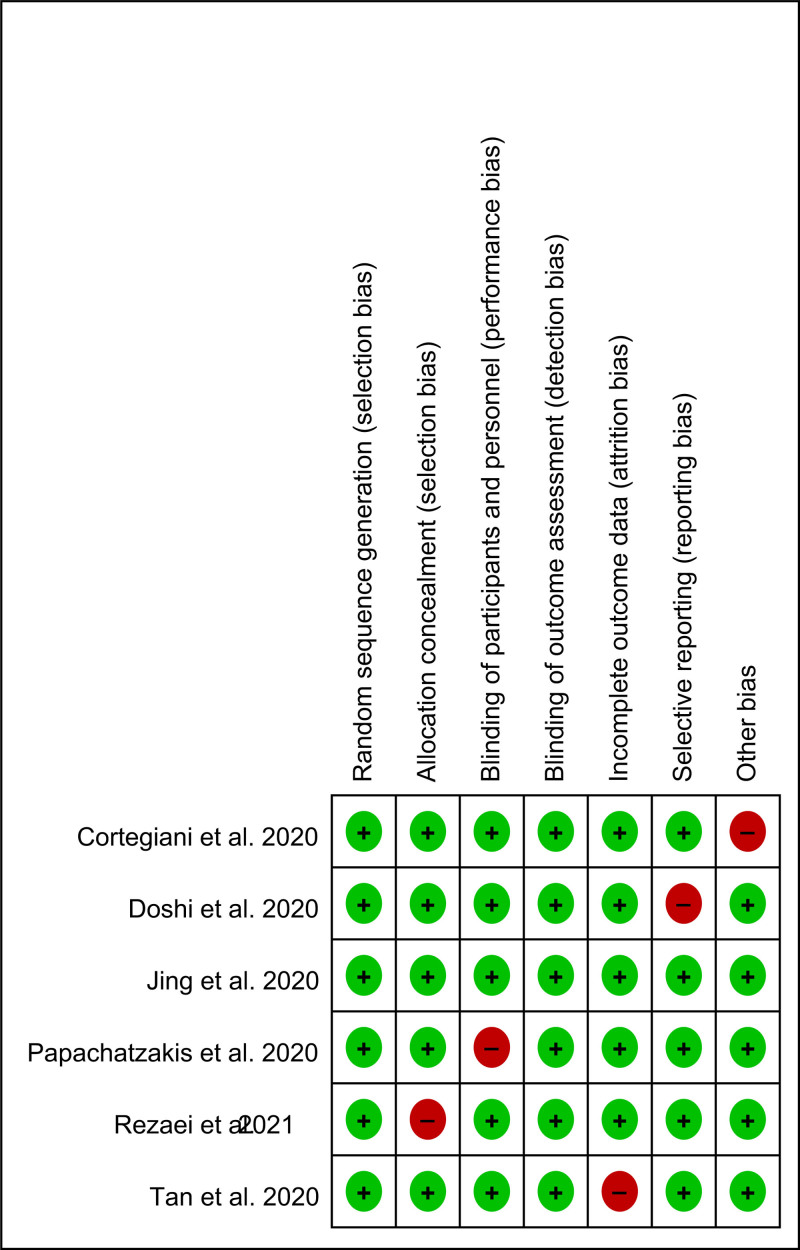
Risk of bias assessment for the included randomized controlled trials using the Cochrane RoB 2.0 tool. RoB = Risk of Bias.

Most studies demonstrated a low risk of bias in key domains, including random sequence generation, allocation concealment, blinding of participants and personnel, blinding of outcome assessment, incomplete outcome data, and selective reporting. However, a small number of studies exhibited potential concerns in certain domains, particularly in allocation concealment, blinding procedures, and reporting bias. Additionally, some studies were judged to have an unclear or high risk of other bias. Despite these limitations, the majority of the evidence was considered to be of moderate-to-high methodological quality, supporting the reliability of the pooled results (Fig. 2).

### 3.4. Partial pressure of carbon dioxide

**Figure 3.**
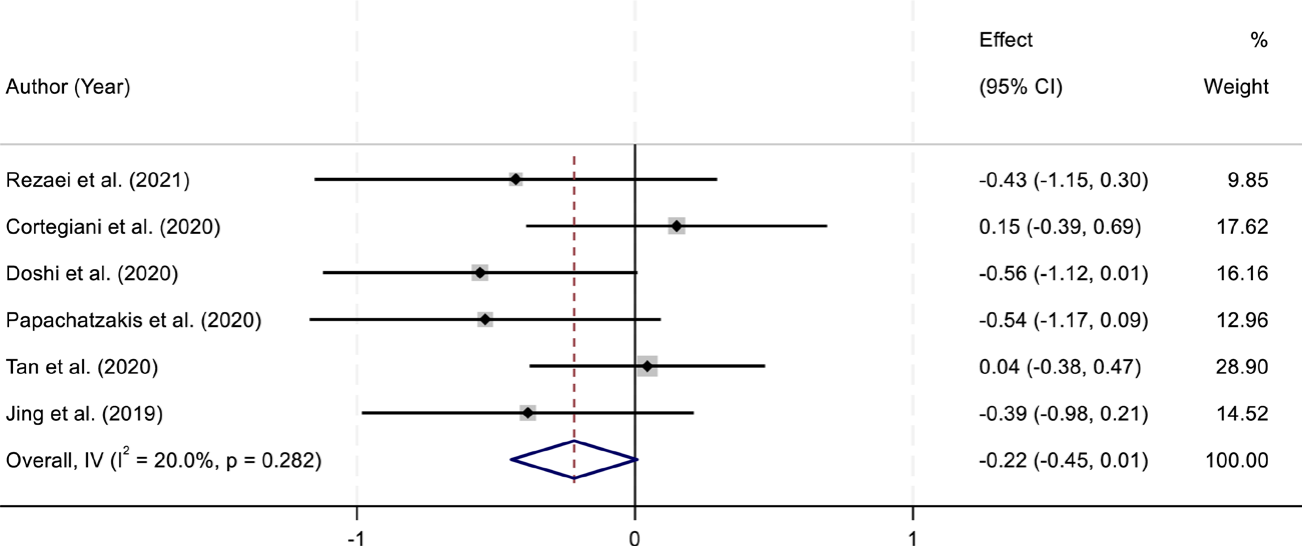
Forest plot comparing the effects of high-flow nasal cannula (HFNC) and noninvasive ventilation (NIV) on partial pressure of carbon dioxide (PaCO_2_). CI = confidence interval, HFNC = high-flow nasal cannula, NIV = noninvasive ventilation, PaCO_2_ = partial pressure of carbon dioxide.

Six RCTs reported measurements of PaCO_2_ in patients receiving HFNC or NIV. Heterogeneity analysis indicated no significant variability among the included studies (*I*^2^ = 20.0%, *P* = .282). Given the low heterogeneity, a fixed-effect model was applied to synthesize the results. The pooled analysis demonstrated that there was no statistically significant difference in PaCO_2_ levels between the HFNC and NIV groups (SMD = –0.22, 95% CI: –0.45 to 0.01; *P* > .05). These findings suggest that HFNC provides a comparable effect to NIV in terms of CO_2_ clearance in patients with AHRF. The consistency across studies strengthens the reliability of this conclusion (Fig. 3).

### 3.5. Arterial pH

**Figure 4.**
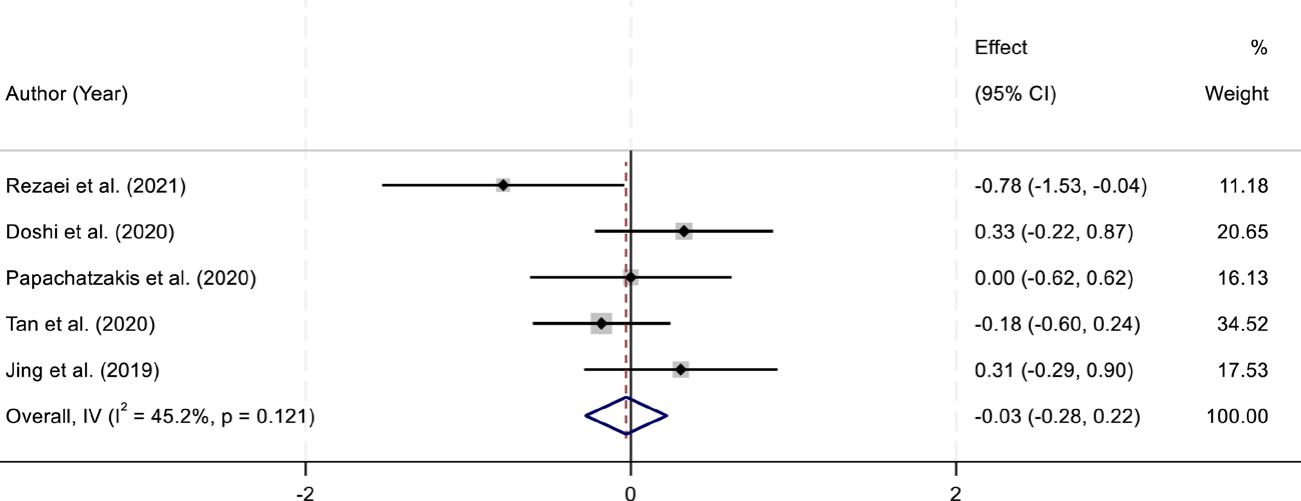
Forest plot comparing the effects of HFNC and NIV on arterial pH levels in patients with acute hypercapnic respiratory failure. CI = confidence interval, HFNC = high-flow nasal cannula, NIV = noninvasive ventilation.

Five RCTs reported arterial pH measurements in patients treated with HFNC compared with NIV. Assessment of heterogeneity revealed no significant inconsistency across the included studies (*I*^2^ = 45.2%, *P* = .121). Given the moderate but nonsignificant heterogeneity, a fixed-effect model was applied for data synthesis. The pooled results indicated that there was no statistically significant difference in arterial pH between the HFNC and NIV groups (SMD = –0.03, 95% CI: –0.28 to 0.22; *P* > .05). These findings suggest that both interventions achieved a comparable effect in correcting acidosis during AHRF (Fig. 4).

### 3.6. Intubation

**Figure 5.**
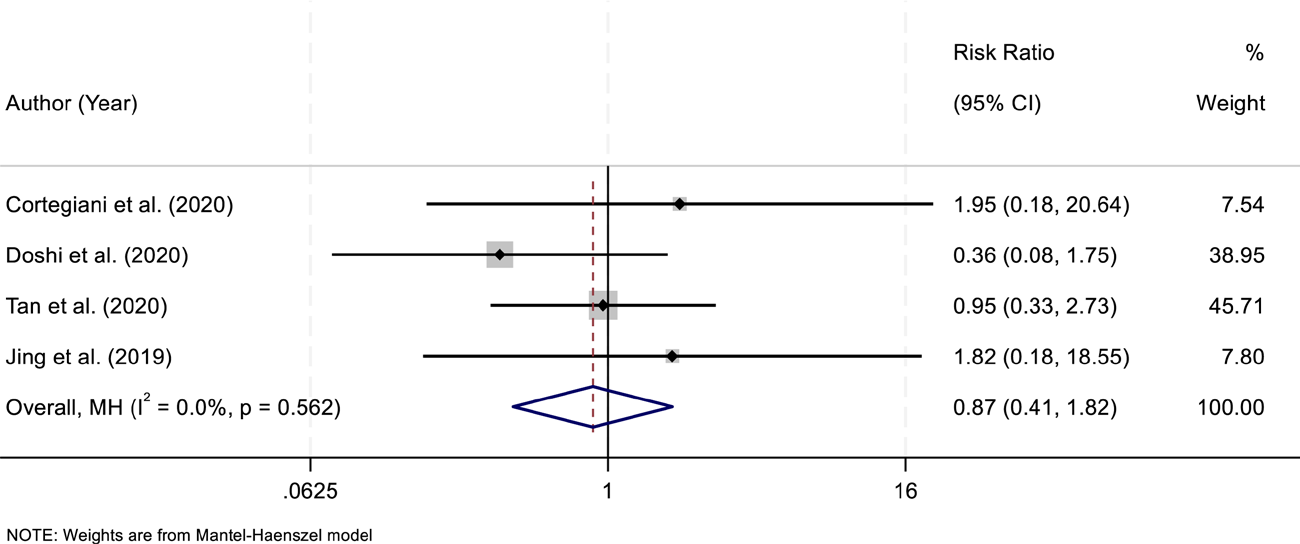
Forest plot comparing the risk of endotracheal intubation between HFNC and NIV groups. CI = confidence interval, HFNC = high-flow nasal cannula, NIV = noninvasive ventilation.

Four RCTs evaluated the requirement for endotracheal intubation among patients treated with HFNC compared with NIV. Heterogeneity analysis indicated no significant variability across the included studies (*I*^2^ = 0.0%, *P* = .562). Based on this low heterogeneity, a fixed-effect model was applied to pool the data. The meta-analysis demonstrated that there was no statistically significant difference in the risk of intubation between HFNC and NIV groups (RR = 0.87, 95% CI: 0.41–1.82; *P* > .05). These findings suggest that HFNC and NIV offer comparable efficacy in preventing intubation in patients with AHRF (Fig. 5).

### 3.7. Mortality

**Figure 6.**
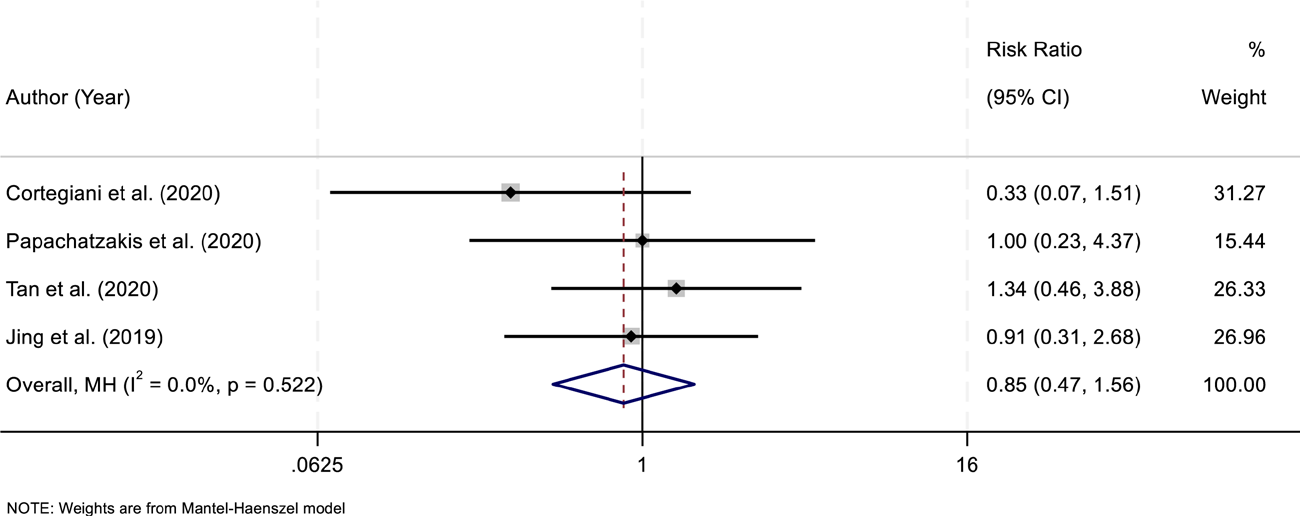
Forest plot comparing mortality outcomes between patients treated with HFNC and those treated with NIV. CI = confidence interval, HFNC = high-flow nasal cannula, NIV = noninvasive ventilation.

Four RCTs reported data on mortality in patients receiving HFNC compared with those treated with NIV. Analysis of heterogeneity demonstrated no significant inconsistency among the included studies (*I*^2^ = 0.0%, *P* = .522). Given the absence of heterogeneity, a fixed-effect model was employed to synthesize the results. The pooled analysis showed no statistically significant difference in mortality between the HFNC and NIV groups (RR = 0.85, 95% CI: 0.47–1.56; *P* > .05). These findings indicate that HFNC provides a survival outcome comparable to that of NIV in patients with AHRF (Fig. 6).

### 3.8. Publication bias

Publication bias was evaluated using Egger’s linear regression test. The analysis showed no significant evidence of publication bias among the included studies (*P* > .05 for all). These findings suggest that the pooled estimates were not substantially influenced by small-study effects, supporting the robustness of the results.

## 4. Discussion

This meta-analysis synthesized RCT evidence comparing HFNC with NIV for the initial management of AHRF. Across continuous physiologic endpoints and patient-centered outcomes, pooled estimates indicated no statistically significant differences between HFNC and NIV in the magnitude of PaCO_2_ reduction, correction of arterial pH, risk of endotracheal intubation, or all-cause mortality. Statistical heterogeneity was low to absent for most outcomes, supporting the internal consistency of the pooled effects. Taken together, the findings suggest that, in the trial populations studied, HFNC achieved gas-exchange and short-term clinical outcomes comparable to NIV, the guideline-endorsed standard of care for hypercapnic decompensation, particularly in exacerbations of COPD.

From a physiological perspective, the neutrality of effects across PaCO_2_ and pH is mechanistically plausible. HFNC provides heated, humidified high flows that generate a modest pharyngeal positive end-expiratory pressure and washout of nasopharyngeal dead space, thereby improving ventilatory efficiency and reducing inspiratory effort. NIV, in contrast, augments alveolar ventilation more explicitly by delivering pressure support and adjustable positive end-expiratory pressure. The comparable net effects observed here likely reflect the balance between HFNC’s dead space washout and comfort advantages and NIV’s direct augmentation of minute ventilation.^[19,20]^ The absence of a differential signal in intubation or mortality also aligns with this physiologic equipoise, as both modalities can stabilize gas exchange and reduce work of breathing when applied early, monitored closely, and escalated promptly when the initial strategy fails.

The modest and statistically non-significant pooled standardized mean differences for PaCO_2_ and pH merit cautious interpretation. Most included trials enrolled patients with mild-to-moderate acidemia and hypercapnia, populations in whom the relative advantage of aggressive ventilatory assistance may be attenuated. In more severe respiratory acidosis – where elevated ventilatory drive, dynamic hyperinflation, and respiratory muscle fatigue are prominent – NIV’s ability to ensure adequate alveolar ventilation could be expected to yield greater PaCO_2_ clearance. Conversely, in patients with significant interface intolerance or secretion burden, HFNC’s comfort and airway-conditioning benefits may sustain adherence and permit adequate ventilatory unloading. The net effect at the population level thus trends toward equivalence when the spectrum of severity is broad and crossovers or rescue strategies are permitted.^[21,22]^

The observed equivalence in endotracheal intubation risk and mortality must also be placed in the context of trial protocols that allowed crossover or escalation per predefined clinical triggers. Early recognition of non-response, structured monitoring, and timely transition from HFNC to NIV (or vice versa) are central to avoiding delayed intubation. In such pragmatic frameworks, either modality may serve as an initial step while maintaining a low threshold for escalation. Importantly, the low between-study heterogeneity for intubation and mortality suggests that protocolized rescue strategies were effective in preventing downstream harm from initial modality selection.

Our synthesis accords with and extends multiple analyses and trials published within the past 3 years. A 2023 systematic review and meta-analysis focusing on AHRF reported no significant differences between HFNC and NIV for correction of PaCO_2_, pH, or PaO_2_ and found no differences in intubation or mortality – results that mirror the present findings and add convergent evidence that, at a population level, HFNC and NIV yield broadly similar outcomes when used as first-line noninvasive support in AHRF settings.^[23]^ However, a 2024 single-center, unblinded, non-inferiority RCT in acute exacerbation of chronic obstructive pulmonary disease (AECOPD) with moderate hypercapnic acute respiratory failure concluded that HFNC was not non-inferior to NIV for the composite of treatment failure and showed higher intubation rates and less PaCO_2_ reduction at 48 hours with HFNC, despite better comfort and fewer skin-related complications. This trial highlights that, in more acidemic COPD exacerbations requiring sustained ventilatory unloading, NIV may provide superior control of hypercapnia over time.^[24]^

Narrative and scoping reviews published in 2024 have synthesized the physiologic rationale and emerging clinical data, proposing that while HFNC and NIV may demonstrate similar overall clinical success in hypercapnic AHRF, NIV appears more consistently effective for rapid PaCO_2_ reduction whereas HFNC confers advantages in comfort, secretion management, and tolerance. These reviews also emphasize technique-dependent considerations (e.g., cannula size, flow settings) that can influence CO_2_ washout and the trade-off between washout and end-expiratory pressure.^[25]^ More recently, meta-analytic work in 2025 restricted to AECOPD with hypercapnic ARF similarly suggested comparable treatment success between HFNC and NIV, with a tendency for NIV to achieve faster early physiologic improvements and HFNC to demonstrate better tolerability. In network meta-analytic frameworks comparing multiple noninvasive strategies, the relative ranking likewise favored NIV for ventilatory targets while acknowledging HFNC’s usability advantages. These complementary analyses underscore the importance of patient selection, early reassessment, and structured escalation algorithms rather than a 1-size-fits-all preference.^[26]^ Overall, the alignment between our pooled results and recent literature suggests that, in unselected or moderately acidemic AHRF populations, HFNC can serve as a viable initial modality without compromising key outcomes, provided that clinicians maintain vigilant monitoring and implement predefined criteria for escalation, particularly when hypercapnia does not improve promptly.

For front-line practice, these results support HFNC as a feasible alternative to NIV for initial noninvasive support in AHRF when NIV is not tolerated, contraindicated, or logistically delayed, with the proviso that escalation thresholds are explicit and time-bound. NIV should remain the preferred first-line option for patients with more severe acidemia or when rapid, sustained PaCO_2_ reduction is prioritized. Protocols should incorporate early reassessment (e.g., within 1–2 hours), objective failure criteria, and rapid transition to NIV or invasive ventilation when predefined targets are unmet.^[18]^

A key strength of this work is the focus on RCTs, thereby minimizing selection and confounding biases inherent to observational designs. The consistency of findings across outcomes, coupled with low heterogeneity for intubation and mortality, enhances the credibility of the pooled estimates. The use of standardized risk-of-bias assessment and prespecified analytic choices (fixed-effect models when heterogeneity was low) supports methodological rigor. Furthermore, publication bias was not detected using Egger’s linear regression test, reducing concern for small-study effects influencing the conclusions. Nevertheless, several limitations warrant consideration. First, most included trials enrolled patients with mild-to-moderate acidosis, limiting inference for those with severe hypercapnia and pH < 7.30, in whom NIV’s ventilatory support may be more decisively beneficial. Second, trials were typically open-label and allowed crossover or rescue, which is clinically appropriate but may attenuate between-group differences in patient-centered outcomes. Third, co-interventions, escalation criteria, and treatment protocols (e.g., HFNC flow and cannula size, NIV interface and pressure settings) varied across studies; these pragmatic variations may dilute modality-specific effects and constrain external validity to settings with similar protocols and staffing expertise. Fourth, several trials were single-center with modest sample sizes, restricting precision for rare outcomes and limiting subgroup exploration. Fifth, because the included studies were conducted across emergency departments, intensive care units, and respiratory units with different staffing and monitoring capacities, context-sensitive factors (e.g., expertise in NIV titration or adherence support) could have influenced outcome trajectories. Finally, while our results align with recent systematic reviews and targeted RCTs, the evidence base remains underpowered to rule out small but clinically meaningful differences in selected subgroups (e.g., severe acidemia, obesity hypoventilation, or neuromuscular etiologies).

In aggregate, the available randomized evidence indicates that HFNC and NIV produce broadly comparable early gas-exchange corrections and short-term clinical outcomes in AHRF, with equipoise for intubation and survival at the population level. The literature from the last 3 years provides nuance: NIV may better sustain PaCO_2_ reductions over longer intervals in acidemic AECOPD, while HFNC confers comfort and airway-conditioning benefits that can improve tolerance and secretion management. Optimizing the initial choice may therefore hinge less on a universal hierarchy and more on acuity, trajectory of PaCO_2_/pH in the first hours, patient tolerance, secretion burden, and local competence with either modality. Operationalizing this approach requires explicit early reassessment windows, failure criteria (e.g., predefined PaCO_2_ or pH targets and clinical signs of fatigue), and streamlined escalation pathways. Priorities for future trials include multicenter, adequately powered RCTs that stratify by baseline acidosis severity and COPD phenotype, standardize HFNC flows and cannula sizing, and apply uniform NIV titration targets. Patient-important outcomes – comfort, device tolerance, skin integrity, secretion clearance, and work of breathing – should be captured alongside gas-exchange endpoints and escalation metrics. Adaptive protocols that embed real-time switching rules could test whether algorithm-driven transitions improve efficacy and safety compared with fixed initial assignments. Finally, the role of HFNC as a bridge for NIV-intolerant patients, or as an adjunct between NIV sessions, should be evaluated in pragmatic designs reflecting real-world practice.

## 5. Conclusions

This meta-analysis demonstrated that HFNC and NIV provide comparable outcomes in patients with AHRF. No significant differences were observed in PaCO_2_ reduction, arterial pH correction, intubation risk, or mortality. These findings suggest that HFNC may serve as a feasible alternative to NIV, provided that careful patient selection and timely escalation strategies are applied.

## Author contributions

**Conceptualization:** Cong Chen, Yanfang Peng, Jiahui Li, Shibin Peng.

**Data curation:** Cong Chen, Yanfang Peng, Jiahui Li, Shibin Peng.

**Formal analysis:** Cong Chen, Yanfang Peng, Jiahui Li, Shibin Peng.

**Funding acquisition:** Cong Chen, Shibin Peng.

**Investigation:** Cong Chen.

**Writing** – **original draft:** Cong Chen, Yanfang Peng, Jiahui Li.

**Writing** – **review & editing:** Cong Chen, Yanfang Peng, Jiahui Li.
